# Usefulness of ultrasonography for diagnosing iatrogenic spinal accessory nerve palsy after lymph node needle biopsy: a case report

**DOI:** 10.1186/s12891-020-03737-w

**Published:** 2020-10-31

**Authors:** Hisataka Suzuki, Yuichiro Matsui, Takahito Iwai, Mutsumi Nishida, Norimasa Iwasaki

**Affiliations:** 1 Department of Orthopaedic Surgery, Faculty of Medicine, Graduate School of Medicine, Hokkaido University, Kita 15, Nishi 7, Kita-ku, Sapporo, Hokkaido 060-8648 Japan; 2 Division of Laboratory and Transfusion Medicine/Diagnostic Center for Sonography, Hokkaido University Hospital, Kita 14, Nishi 5, Kita-ku, Sapporo, Hokkaido 060-8648 Japan

**Keywords:** Ultrasonography, Spinal accessory nerve palsy, Iatrogenicity, Needle biopsy, Neurolysis

## Abstract

**Background:**

Spinal accessory nerve (SAN) palsy is rare in clinical settings. Iatrogenicity is the most common cause, with cervical lymph node biopsy accounting for > 50% of cases. However, SAN palsy after lymph node needle biopsy is extremely rare, and the injury site is difficult to identify because of the tiny needle mark.

**Case presentation:**

A 26-year-old woman was referred to our hospital with left neck pain and difficulty abducting and shrugging her left shoulder after left cervical lymph node needle biopsy. Five weeks earlier, a needle biopsy had been performed at the surgery clinic because of suspected histiocytic necrotizing lymphadenitis. No trace of the needle biopsy site was found on the neck, but ultrasonography (US) showed SAN swelling within the posterior cervical triangle. At 3 months after the injury, her activities of daily living had not improved. Therefore, we decided to perform a surgical intervention after receiving informed consent. We performed neurolysis because the SAN was swollen in the area consistent with the US findings, and nerve continuity was preserved. Shoulder shrugging movement improved at 1 week postoperatively, and the trapezius muscle manual muscle testing score recovered to 5 at 1 year postoperatively. The swelling diameter on US gradually decreased from 1.8 mm preoperatively to 0.9 mm at 6 months.

**Conclusion:**

We experienced a rare case in which US was useful for iatrogenic SAN palsy. Our results suggest that preoperative US is useful for localization of SAN palsy and that postoperative US for morphological evaluation of the SAN can help assess recovery.

## Background

Spinal accessory nerve (SAN) palsy is rare in clinical settings. Iatrogenicity is the most common cause (70–90%), with cervical lymph node biopsy accounting for > 50% of cases [1, 2]. However, SAN palsy after lymph node needle biopsy is extremely rare. The SAN descends in the neck from the jugular foramen to the anterior border of the upper trapezius muscle and can be damaged at any level. Thus far, iatrogenic SAN palsy has been diagnosed by physical examination, electromyographic findings, and MRI. The injury site can be inferred from the operative scar after cervical surgery. However, the injury site is difficult to identify after lymph node needle biopsy because of the tiny needle mark.

Here, we report an extremely rare case of iatrogenic SAN palsy after lymph node needle biopsy and describe the usefulness of ultrasonography (US) for preoperative localization diagnosis and nerve morphology evaluation. The patient provided written informed consent for publication of this report.

## Case presentation

A 26-year-old woman was referred to our hospital with left neck pain and difficulty in abducting and shrugging her left shoulder. About 5 weeks earlier, she experienced fever of ≥38 °C and cervical lymphadenopathy. Histiocytic necrotizing lymphadenitis was suspected and left cervical lymph node needle biopsy was performed. Subsequently, she became aware of left neck pain and difficulty in left shoulder abduction and shrugging.

**Fig. 1:**
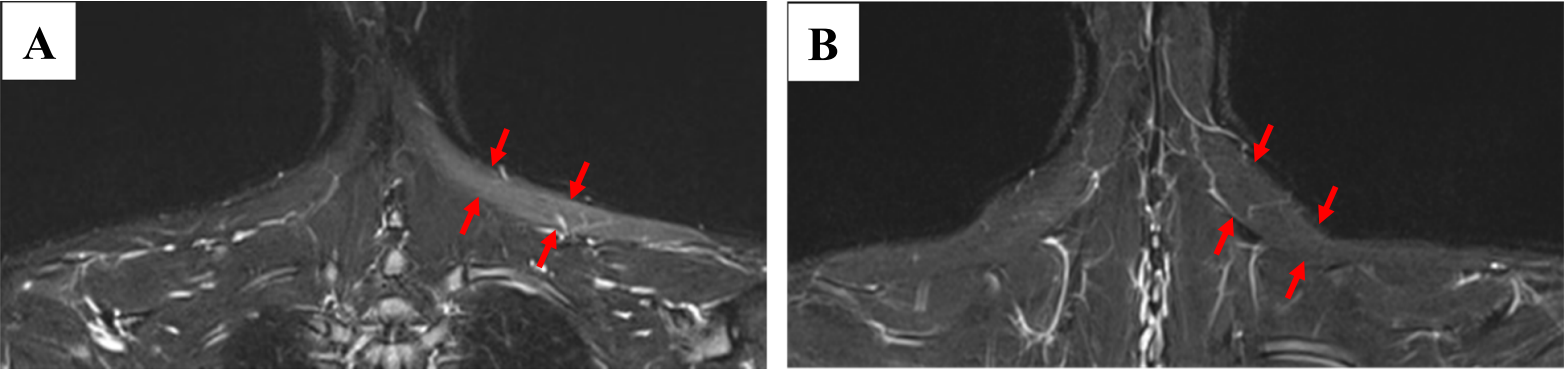
MRI findings. **a** A short T1 inversion recovery image showed high signal intensity in the left trapezius muscle before surgery (*red arrows*). **b** At 1 year postoperatively, the signal strength of the left trapezius muscle was reduced (*red arrows*)

**Fig. 2:**
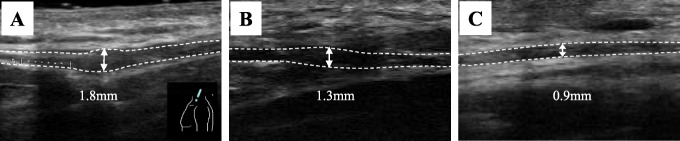
US examination findings. **a** Ultrasonography was performed using a Canon Aplio i800 with a 24-MHz linear probe. Preoperatively, the SAN was swollen (1.8-mm width) at the central sternocleidomastoid muscle level within the posterior cervical triangle, but nerve continuity was preserved. The diameter of the SAN gradually improved from 1.8 mm preoperatively to 1.3 mm at 3 months (**b**) and 0.9 mm at 6 months (**c**), at which time there was no swollen area

Her left shoulder showed mild trapezius muscle atrophy and scapular winging. No trace of the needle biopsy was found on the neck and Tinel’s sign was not observed. Shoulder range of motion (ROM) was 100° for flexion and 100° for abduction. The manual muscle testing (MMT) scores were 5 for the sternocleidomastoid muscle and 2 for the upper trapezius muscle. On electromyography, the upper, middle, and lower trapezius muscle amplitudes decreased during strong contraction. Magnetic resonance imaging (MRI) showed atrophy of all three left trapezius muscle regions (Fig. 1). US was performed with a clinical ultrasound system (Aplio i800; Canon Medical Systems, Otawara, Japan) using a 24-MHz linear probe. We initially identified the external jugular vein by a short-axis US image. The SAN was then identified in the posterior cervical triangle on the dorsal side of the external jugular vein and under the sternocleidomastoid muscle. US showed SAN swelling (1.8-mm width) at the central sternocleidomastoid muscle level within the posterior cervical triangle, but nerve continuity was preserved (Fig. 2). At 3 months after the injury, shoulder ROM showed slight improvement with flexion of 140° and abduction of 140°, but the MMT score and activities of daily living such as placing things on a shelf and using a hair dryer, did not improve. Therefore, we decided to perform a surgical intervention after receiving informed consent.

**Fig. 3:**
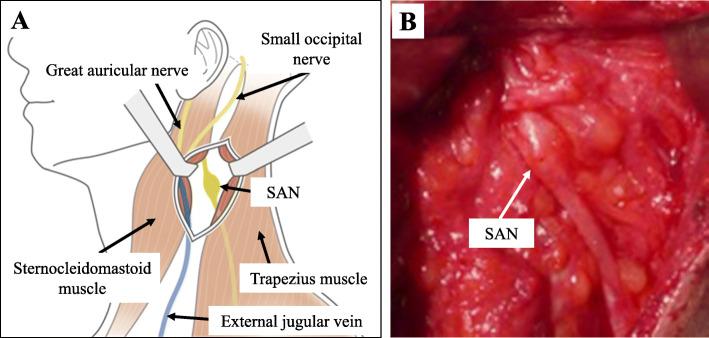
Intraoperative findings. **a** A skin incision was made along the posterior sternocleidomastoid muscle edge. **b** The SAN was swollen in the area consistent with the US findings and highly adherent to surrounding tissues, but nerve continuity was preserved

An approximately 5-cm skin incision was made along the posterior sternocleidomastoid muscle edge, centering on the SAN swelling identified on the preoperative US image, and the surgical site was carefully approached. The SAN was exposed, avoiding the external jugular vein and retracting the sternocleidomastoid muscle. The SAN was swollen in the area consistent with the US findings and highly adherent to the surrounding tissues, but the nerve continuity was preserved (Fig. 3). Intraoperative electrical stimulation of the nerve evoked trapezius muscle contraction. Therefore, we performed neurolysis of the SAN. Postoperative rehabilitation was performed without movement restrictions.

Shoulder shrugging movement improved at 1 week postoperatively and ROM recovered after 1 month. The trapezius muscle MMT score recovered to 5 at 1 year postoperatively. Amplitude on electromyography was increased at 3 months. The diameter of the SAN on US was gradually reduced from 1.8 mm preoperatively to 1.3 mm at 3 months and 0.9 mm at 6 months, at which time no swollen area could be identified (Fig. 2). The SAN could be easily identified by applying a probe centered on the operation scar. At 1 year, MRI showed improved signal strength of the left trapezius muscle (Fig. 1). At 2 years, she could smoothly move her left shoulder almost completely without pain.

## Discussion and conclusion

Many SAN palsy cases are reported to be iatrogenic [2]. During cervical surgery, it is always necessary to consider SAN injury. However, SAN damage is often not noticed immediately, and the diagnosis is delayed [2, 3]. Early diagnosis and surgery within 3–6 months are recommended when improvement is poor [3–5]. Physical findings, electrophysiology, and MRI are useful for early diagnosis of SAN palsy [6]. In our case, we diagnosed SAN palsy. However, we performed surgery at 3 months after injury because her condition showed poor improvement.

It is often difficult to identify the damaged area in iatrogenic nerve palsy, particularly in cervical lymph node needle biopsy cases that often have no trace of the needle insertion point. In 2002, Bodner et al. [6] described the usefulness of US for management of three patients with iatrogenic SAN palsy. Subsequently, Cesmebasi et al. [7] and Shen et al. [8] reported that preoperative US visualized nerve transection in some cases with iatrogenic SAN palsy. We also identified the damaged area of the SAN on preoperative US. However, our case was characterized by poor findings on visual inspection and palpation after cervical needle biopsy. Seddon’s classification [9] divides nerve injuries into three categories (neurapraxia, axonotmesis, and neurotmesis), providing a basis for establishing the prognosis and determining the potential treatment strategy. Neurotmesis can be identified by observing the nerve stumps by US. If pseudoneuroma formation is observed, axonotmesis is considered to be present. However, it is difficult to differentiate neuropraxia from axonotmesis when nerve malformation is not observed. In our case, US findings showed pseudoneuroma formation and continuity of the SAN, and the nerve injury was considered to be neurotmesis according to Seddon’s classification. The intraoperative findings of the nerve were similar, and trapezius muscle contraction was observed on intraoperative electrical stimulation. Because SAN swelling was detected on US, we were able to identify the damaged area and plan the surgery. In iatrogenic SAN palsy cases with difficulty in estimating the damaged area by visual inspection or palpation, preoperative US may enable minimally invasive surgery. To prevent iatrogenic SAN palsy, we believe that it is necessary to consider the risk of SAN palsy and identify the SAN using US when performing a cervical lymph node biopsy.

In 2016, Göransson et al. [5] reported good results for neurolysis when contraction of the dominant muscle was observed on nerve stimulation, and for nerve repair using sutures or nerve transplantation when contraction was not observed. In our case, US and intraoperative findings showed that nerve continuity was preserved, and trapezius muscle contraction was observed on intraoperative electrical stimulation. Therefore, we performed neurolysis of the SAN.

In 2019, Li et al. [10] reported postoperative morphological recovery of the median nerve in carpal tunnel syndrome observed using US. The average diameter of the swollen area of the median nerve was 0.258 cm at 2 weeks postoperatively, but subsequently improved to 0.225 cm at 3 months and 0.214 cm at 12 months, and reached the normal range. They reported that the degrees of improvement in clinical symptoms were large when US showed a tendency toward swelling improvement at 3 months postoperatively. However, the detailed postoperative morphological changes in the SAN palsy were not reported. In our case, it required about 1 year to recover the strength of the upper trapezius muscle. However, at 3 months postoperatively, it was considered that further alleviation of the clinical symptoms could be expected because US showed morphological improvement of the SAN. It is possible to confirm the degree of postoperative improvement of the SAN neuropathy by electromyography. Thus, postoperative morphological evaluation of the SAN by US may be a useful noninvasive technique to detect recovery from postoperative neuropathy.

We experienced a case in which US was useful for iatrogenic SAN palsy. Our results suggest that preoperative US is useful for localization of SAN palsy and that postoperative US for morphological evaluation of the SAN is one of the indicators of recovery.

## Data Availability

All data concerning the case are presented in the manuscript.

## Abbreviations

SAN: Spinal accessory nerve
US: Ultrasonography
ROM: Range of motion
MMT: Manual muscle testing
MRI: Magnetic resonance imaging
